# Mentoring Students with Intellectual and Developmental Disabilities: Evaluation of Role-Specific Workshops for Mentors and Mentees

**DOI:** 10.1007/s10803-020-04599-w

**Published:** 2020-07-14

**Authors:** Rumi Agarwal, Laura Heron, Mitra Naseh, Shanna L. Burke

**Affiliations:** 1 BRAINN Lab, Robert Stempel College of Public Health and Social Work, Florida International University, 11200 S.W. 8th Street, Miami, FL 33199 USA; 2 FIU Embrace, Florida International University, 11200 S.W. 8th Street, Miami, FL 33199 USA; 3 Department of Health Promotion and Disease Prevention, Robert Stempel College of Public Health and Social Work, Florida International University, 11200 S.W. 8th Street, Miami, FL 33199 USA; 4 Department of Industrial and Organizational Psychology, Florida International University, 11200 S.W. 8th Street, Miami, FL 33199 USA; 5 School of Social Work, Robert Stempel College of Public Health and Social Work, Florida International University, 11200 S.W. 8th Street, Miami, FL 33199 USA

**Keywords:** Mentor, Mentee, Workshop, Intellectual disability, Developmental disability, Mentoring

## Abstract

Transitioning to post-secondary education is often challenging for students with intellectual and developmental disabilities (IDD). To address this, Florida International University, specifically FIU Embrace, piloted the Embrace Mentoring Program (EMP), which provided unique role-specific workshops to both faculty/staff mentors, and student mentees with IDD. A mixed-method design was used to analyze knowledge acquisition and participant perceptions of the workshops. Quantitative findings indicated knowledge improvement in a key area for mentors, while qualitative data demonstrated a positive response to workshop content, and highlighted areas of improvement for future workshops. Ultimately, the results from the pilot EMP demonstrated promise in supporting students with IDD towards academic and career-related goals, by providing mentorship training to both mentors *and* mentees.

The Higher Education Opportunity Act (HEOA; United States Department of Education 2010) and funding from grants such as the Transition and Postsecondary Programs for Students with Intellectual Disability (TPSID; Department of Education) have created opportunities for students with disabilities to pursue post-secondary education. These programs are intended to provide inclusive academic college courses, enhance social and personal engagement, and improve employment outcomes (United States Department of Education 2017). The positive effect of higher levels of educational attainment on employment outcomes for individuals with a disability is evident. For example, individuals with a disability who complete some type of college degree, or receive an associate, bachelor’s or higher degree, are more likely to be employed (21.8–28.5%), compared to those with lower levels of education (9.8–15.6%; Bureau of Labor Statistics 2019). Unfortunately, study completion and graduation rates of students with disabilities are lower (41%) than post-secondary students without disabilities (52%; Newman et al. 2011). This disparity has been attributed to the significant challenges students with disabilities encounter during the transition to post-secondary institutes, which often results from poor self-advocacy and self-determination skills (Stodden and Conway 2000), low academic self-efficacy (Pajares 1996), and limited resources and support (Lloyd 2015).

Several studies show evidence that adult and peer mentor programs are positive support mechanisms for students with intellectual and developmental disabilities (IDD; Ames et al. 2016; Culnane et al. 2016; Curtin et al. 2016; Diener et al. 2016; Dipeolu et al. 2015; Eisenman and Freedman 2017; Hotez et al. 2018; Jones and Goble 2012). Having access to and developing relationships with adults such as faculty members, and participating in meetings focused on advising and counseling have shown to be important practices which can support students with IDD, such as those diagnosed with autism spectrum disorders (ASD; Brown and Coomes 2016).

However, an effective mentoring relationship requires mentor and mentee competence in role-specific knowledge and skills, which can otherwise impede desired student outcomes. Therefore, mentoring programs must holistically address areas of potential deficiency by providing training to both mentors and mentees (Jones and Goble 2012; Pfund et al. 2006; Taylor 2003). For faculty and staff mentors to be effective in helping their mentees with disabilities, for example, it is necessary to acquire competence in disability awareness, and specific strategies to support students in developing and accomplishing academic goals (Brown et al. 2010). Further, mentees with IDD require training in soft skills (which is an existing deficit in many disability diagnoses, such as ASD), including understanding formal business etiquette, and recognizing or understanding communication and social cues (Brown et al. 2010; Dipeolu et al. 2015). Finally, both mentors and their mentees with IDD would benefit from an initial orientation session (Jones and Goble 2012), where they can meet, and engage in ice-breaker activities.

Unfortunately, despite the positive outcomes associated with mentoring programs, there is limited literature examining the transition experience to post-secondary education specifically for students with IDD. Furthermore, to date, no studies have addressed the need for training *both* mentors and mentees for their respective roles. To address this gap, a TPSID institution nested within a large public university in South Florida, developed, implemented and evaluated the pilot Embrace Mentoring Program (EMP). The aims of the EMP were to 1) design a mentoring program supporting weekly dyad meetings, 2) develop role-specific workshops to enhance knowledge of faculty/staff mentors, 3) develop role-specific workshops to enhance knowledge of student mentees with IDD, and 4) evaluate the program by assessing knowledge change before and after each workshop, and examine participant feedback to workshops. This paper presents a summary of aims 1, 2, and 3 with a specific focus on examining findings for aim 4. The research questions examined in this paper include: 1) Did mentors and mentees demonstrate knowledge acquisition through attendance in mentoring workshops, and 2) What are the perceptions and feedback from mentors and mentees regarding EMP workshops?

## Methods

### Faculty and Staff Mentors

Using a convenience sample of faculty and staff available on the Florida International University campus, participants from across several departments (n = 31) were recruited voluntarily using flyers and emails distributed throughout campus during summer 2018. Of all recruited mentors, 74% were females (n = 23), and 77% of mentors were employed in a staff/administrative capacity on the university campus (n = 24). Informed consents detailing a two-semester commitment (one academic year) were obtained and background checks were completed. Mentors received a stipend of $400 each semester. Mentors were required to: 1) meet with their assigned  student mentee once a week for an hour on campus, with a specific focus on helping mentees work towards academic and career goals as identified in the student’s STAR (Students Transitioning to Adult Roles) Person-Centered-Plan (PCP), which is explained in detail later (Hayes and Muldoon 2013; aim 1 of the EMP), and 2) attend six mentoring workshops as scheduled throughout the academic year (aim 2 of the EMP).

### Student Mentees

All students with IDD enrolled in the EMP (n = 35) during fall 2018 and spring 2019, consented to participate in the mentoring program and this study, and were matched with a faculty or staff mentor (four mentors were assigned two mentees, respectively, to ensure each student had a mentor). Mentees were also required to 1) meet with their mentor once a week for an hour on campus (aim 1 of the EMP), and 2) attend six mentoring workshops as scheduled throughout the academic year (aim 3 of the EMP).

### Workshop Topics and Content

Four workshop topics were selected for both mentors and mentees, respectively. This was determined through an extant review of mentoring literature, the needs of students with IDD transitioning from high school to a post-secondary environment, and the goals of the mentoring program. Given the likelihood that many mentors may not have experience with providing mentorship, or have experience working closely with a student with IDD specifically in regards to providing academic and career support, it was imperative that mentors be offered training on four specific topics: Program Basics, Disability Awareness, Essential Mentor Skills, and Communication and Employability.

Content within each workshop topic was also purposefully selected. For example, the decision to include Person-First Language (PFL) versus Identity-First Language (IFL) was made on the premise of a few factors. To a large extent, student mentees in the EMP program have a primary diagnosis of intellectual disability with only a few students presenting with an additional diagnosis of a developmental disability, such as autism. Given that preferences for PFL versus IFL differ within disability groups, with IFL gaining prominence largely within the autistic and deaf communities, and individuals with intellectual disabilities preferring PFL (Ferrigon 2019; Lieboweitz 2015), a decision was made to provide training related to PFL.

Workshop topics and content for student mentees with IDD were designed to enhance communication and self-determination skills (Brown et al. 2010; Dipeolu et al. 2015), as deficits in these areas could impede the mentoring relationship. As a result, workshops for student mentees included: Program Basics, Goal Setting, Essential Mentee Skills, and finally, Communication (Brown et al. 2010; Dipeolu et al. 2015).

Both mentor and mentee workshop topics and content were aligned to ensure that dyads could use and reinforce each other’s knowledge during dyad meetings which would improve the mentoring relationship and enhance outcomes. For example, mentors learned the importance of asking their mentee for eye-contact if they were not, while mentees were taught the importance of giving their mentor eye contact. Please see Table 1 for more information on the content of each workshop.

**Table 1:** List of EMP Workshops

Faculty/staff mentor workshops	Student mentee workshops
Orientation Introduction of dyads, description and scope of the program	
Program basics Expectations, scope, legal obligations, pointers for effective mentoring	Program basics Expectations, scope, benefits of being a mentee, privacy and confidentiality, legal rights, etiquette, rules of the program
Disability awareness Disability laws, Person-First Language, types of disability	Goal setting How to develop SMART goals, the importance of self-determination, the difference between positive and negative self-talk
Essential mentor skills Active listening, building trust, goal setting, giving constructive feedback, managing problems, and opening doors	Essential mentee skills Active listening, showing initiative, the importance of following through
Communication and employability Skills and barriers for communication, career opportunities, resources for employment guidance	Communication Verbal/non-verbal cues, open-ended/close-ended questions, self-advocacy
Closing ceremony Sharing of results, completion certificates	

### Workshop Delivery

At the beginning of the academic year (fall 2018), both mentors and mentees attended an orientation session, which involved the initial introduction of mentoring pairs, ice-breaker activities, a brief description of the program, and an outline of program expectations as suggested by Jones and Goble (2012). In the following ten months (over two academic semesters due to scheduling limitations of campus space and time), mentors and mentees attended workshops that encompassed didactic presentations, role-playing, and small-group activities. At the end of the academic year (spring 2019), dyads attended a closing ceremony where they were each awarded the Embrace Mentoring Program certificate of completion.

Each workshop lasted between one to two hours and was conducted in the following sequence: administration of a pre-knowledge acquisition test, workshop presentation, open discussion/activity, and post-knowledge acquisition test. The matched pre- and post- knowledge acquisition tests developed for this pilot study had ten questions, followed a true/false format (except for eight questions across all the knowledge acquisition tests which used a multiple-choice format) and were available for participants to complete via a link to an online, secure (HIPAA compliant) platform. Pre- and post-tests were conducted at all workshops except the orientation and closing ceremony which were held as joint sessions.

In addition to pre- and post- knowledge acquisition tests, quantitative and qualitative data were collected at the end of each semester (fall 2018 and spring 2019) from both mentors and mentees. The end-of-semester survey for mentors and the end-of-semester survey for mentees were designed to gather feedback on both components of the EMP: dyad meetings and workshops. Closed-ended quantitative questions for mentees included: “I knew what was required to do as a mentee,” “I received enough training through the workshops to be a good mentee,” and “Please rate the quality of the workshops you attended.” The first two questions were rated on a 4-point Likert scale ranging from 1 (Strongly Disagree) to 4 (Strongly Agree), and the latter was rated on a 4-point Likert scale ranging from 1 (Poor) to 4 (Excellent). Closed-ended questions for faculty and staff mentors included: “I was clear about the expectations of my role,” “I received sufficient training through the workshops to fulfill my role,” and “Please rate the value of the workshops you attended.” The first two questions were rated on a 4-point Likert scale ranging from 1 (Strongly Disagree) to 4 (Strongly Agree), and the last question was rated on a 4-point Likert scale ranging from 1 (Poor) to 4 (Excellent). Finally, both mentors and mentees were asked the following qualitative, open-ended questions: 1) “Is there anything we could do to improve the workshops?” 2) “Are there any other areas/topics you would have liked to receive training in?” 3) “Which was the most valuable workshop for you?” and 4) “Which was the least valuable workshop for you?”.

### Dyad Meetings

After dyads attended the orientation session, the weekly hour-long mentoring meetings commenced. These ideally took place at the faculty/staff mentor's office on campus. However, if the mentor did not have an office space that was conducive to the meeting, dyads could meet at a campus location where a professional conversation could be undertaken given the focus on academic and career support.

To ensure that mentors had background information on their mentee, program staff provided them with the academic and employment goals of their respective students identified during the STAR PCP (Hayes and Muldoon 2013) process. The STAR PCP allows students with IDD to identify goals with the support from their families and program staff, at the time of enrollment into the post-secondary program. Mentors had significant flexibility regarding the style and strategies employed during each mentoring session to help the mentee accomplish these goals, and as a result, each dyad relationship was unique. However, common activities during meetings included helping students with emails, follow-up and creation of weekly tasks to ensure progress with the STAR PCP goals, and support with building a resume and interviewing. With simultaneous attendance in mentoring workshops, meetings evolved and strengthened over the academic year with the incorporation of knowledge from workshops such as disability etiquette, active listening, providing and receiving feedback, setting SMART goals, and the utilization of resources related to employment support.

### Data Collection and Analysis

Data were collected via the online survey platform Research Electronic Data Capture (RedCap) and quantitative data were analyzed using Statistical Package for Social Sciences V.20 (SPSS; IBM Corp. 2017) For analyzing knowledge acquisition from each workshop, a degree of correctness score was created by coding the correct answers for each question within each workshop (10 questions per workshop) and generating an average pre-workshop score and a post-workshop score. Eight paired sample *t*-tests were then conducted to examine if knowledge increased from before and after each of the four mentee and four mentor workshops, respectively. The Bonferroni correction was applied to paired sample *t* tests to control for multiple comparisons. Data related to participant feedback on mentoring workshops were analyzed using descriptive statistics, frequencies, and a one-sample *t*-test also using SPSS.

Qualitative data were analyzed using thematic analysis (Braun and Clarke 2006). Investigator triangulation (Denzin 1973) was achieved with two authors who independently coded the data into themes and sub-themes. Discussions were held between the coders to share findings, and address discrepancies. Consensus was attained, and a third coder reviewed the final analysis. If disagreements remained or arose, a fourth coder would have been involved as per the study protocol.

## Results

### Quantitative Data Analysis

#### Mentor and Mentee Pre- and Post- Knowledge Acquisition Tests

For faculty and staff mentors, knowledge significantly increased from before and after the Communication workshop (pre-M = 0.667, post-M = 0.789, *t*(17) = − 2.535, *p* = 0.021) and the Disability Awareness workshop (pre-M = 0.633, post-M = 0.750, *t*(17) = − 4.507, *p* = 0.000). Faculty and staff did not demonstrate a significant knowledge difference between pre- and post- for the Program Basics workshop (pre-M = 0.740, post-M = 0.800, *t*(14) = 0.289, *p* = 0.082) and the Essential Mentor Skills workshop (pre-M = 0.556, post-M = 0.606, *t*(17) = − 1.787, *p* = 0.092). When compared to an adjusted alpha level after applying the Bonferroni correction for multiple comparisons, the communication workshop paired *t*-test was no longer significant (α_adjusted_ = 0.0125).

There was not a significant difference in knowledge acquisition from before and after all mentee workshops: Program Basics (pre-M = 0.523, post-M = 0.530, *t*(25) = − 0.212, *p* = 0.834), Communication (pre-M = 0.716, post-M = 0.708, *t*(24) = 0.289, *p* = 0.775), Goal-Setting (pre-M = 0.849, post-M = 0.850, *t*(25) = − 0.028, *p* = 0.978), and Essential Mentee Skills (pre-M = 0.597, post-M = 0.607, *t*(29) = − 0.372, *p* = 0.712). Please see Table 2 for details on mentor and mentee paired *t*-test results.

**Table 2:** Mentor and mentee paired *t*-test results

	Workshop	Pre-mean (SD)	Post-mean (SD)	Effect size (Cohen’s d)	*t*(*df*)	*p*-value
Faculty and staff mentors	Program basics	.740(.106)	.800(.076)	.654	− 1.871(14)	.082
	Disability awareness	.633(.114)	.750(.079)	1.194	− 4.507(17)	.000*
	Essential mentor skills	.556(.143)	.606(.121)	.378	− 1.787(17)	.092
	Communication	.667(.146)	.789(.096)	.990	− 2.535(17)	.021
Student mentees	Program basics	.523(.166)	.529(.138)	.045	− .212(25)	.834
	Goal setting	.849(.125)	.850(.197)	.005	− .028(25)	.978
	Essential mentee skills	.597(.204)	.607(.249)	.044	− .372(29)	.712
	Communication	.716(.128)	.708(.132)	.062	.289(24)	.775

*p-values compared to adjusted alpha level after Bonferroni correction: α_adjusted_ = .0125

#### End of Semester Questions

Results from the end of semester survey for faculty and staff (n = 52) indicated that in response to the first question, “I was clear about the expectations of my role” (M = 3.35, SD = 0.683), the majority of mentors stated that they strongly agreed with the statement (46.2%), while slightly fewer agreed (42.3%), compared to 11.5% who disagreed. In response to the second question “I received sufficient training through the workshops to fulfill my role” (M = 3.31, SD = 0.544), most agreed (61.5%) or strongly agreed (34.6%) with the statement, compared to 3.8% who disagreed. Finally, when asked to rate the value of the workshops (excellent, good and fair; M = 3.31, SD = 0.612), most mentors rated them as good (53.8%) or excellent (38.5%), compared to 7.7% who rated them as fair. To examine whether participants’ scores differed significantly from the median value, a one-sample *t*-test was conducted (with the median set as 2.5 on the 1–4 Likert scale). For all three end-of-semester mentor questions, responses differed significantly from the median value: “I was clear about the expectations of my role”, mean difference = 0.846, *t*(51) = 8.938, *p* = 0.000, “I received sufficient training through the workshops to fulfill my role”, mean difference = 0.808, *t*(51) = 10.712, *p* = 0.000, and “Please rate the value of the workshops you attended”, mean difference = 0.808, *t*(51) = 9.523, *p* = 0.000. All one-sample *t*-tests were significant when compared to an adjusted alpha level after applying the Bonferroni correction (α_adjusted_ = 0.0167).

Findings from the end-of-semester student mentee (n = 37) survey indicated that in response to the first question “I knew what I was required to do as a mentee” (M = 3.54, SD = 0.558), the majority of mentees strongly agreed (56.8%) or agreed (40.5%) with the statement, compared to 2.7% who disagreed. In response to the second question “I received enough training through the workshops to be a good mentee” (M = 3.51, SD = 0.559), most mentees strongly agreed (54.1%) or agreed (43.2%) with the statement, compared to 2.7% who disagreed. Finally, in response to the question “Please rate the quality of the workshops you attended” (M = 3.46, SD = 0.650), most mentees rated the quality of the workshops as excellent (54.1%) or good (37.8%), compared to 8.1% who responded fair. To examine whether participant scores differed significantly from the median value, a one-sample *t*-test was conducted (with the median set as 2.5 on the 1–4 Likert scale). For all three end-of-semester mentee questions, responses differed significantly from the median value: “I knew what I was required to do as a mentee”, mean difference = 1.014, *t*(36) = 11.353, *p* = 0.000, “I received enough training through the workshops to be a good mentee”, mean difference = 1.014, *t*(36) = 11.032, *p* = 0.000, and “Please rate the quality of the workshops you attended”, mean difference = 0.959, *t*(36) = 8.985, *p* = 0.000. All one-sample *t*-tests were significant when compared to an adjusted alpha level after applying the Bonferroni correction (α_adjusted_ = 0.0167).

### Qualitative Data Analysis

Responses to the open-ended questions from the end of semester survey resulted in three major themes across mentor and mentee feedback regarding workshop elements. The three themes identified were workshop content, workshop-style, and logistics.

#### Workshop Content

Mentors and mentees offered substantial feedback related to workshop content, which was defined as the material, knowledge, and/or skills being delivered at the session. Faculty and staff mentor responses indicated that while many mentors found all workshops useful, Disability Awareness, Communication, and Essential Mentor Skills were the most beneficial. Mentors agreed in the need for additional disability training, which could be applied directly to their weekly dyad meetings. Specifically, some mentors expressed a desire to learn how to motivate, develop tasks and exercises, communicate, and understand their mentees better within the limitations mentees may face as a result of their disability.

Feedback on additional resources that would be helpful was also identified. An overwhelming number of mentors stated a need to know their mentees' specific disability, which would allow them to understand their abilities better. In addition, mentors requested information related to the curriculum and schedule of their mentee, a mentor handbook with do’s and don’ts, and recommended books to read on mentoring or counseling. Mentors also wished to learn and understand the STAR PCP (Hayes and Muldoon 2013) process better and sought assistance with ideas for activities that align with these goals.

#### Workshop Style

This second theme was defined as the instructional technique used to deliver the workshop content. Overwhelmingly, the need for increased interaction within and between mentor and mentee groups was evident. Responses highlighted that the orientation session which integrated mentors and mentees was the most valued by both groups. In addition, the need for an active learning style during workshops was also expressed by mentors and mentees. Specifically, suggestions included more open forum time, and for workshops to be more dynamic, active, and interactive.

#### Workshop Logistics

The final theme related to the logistics of workshop facilitation emerged. In reference to scheduling, the dates and times of the workshops seemed to conflict with the availability of many mentors. As such, it was suggested to offer workshops through an online format. In addition, most mentors commented on the need for workshops to be offered earlier in the fall semester or even offered before the official start of the mentoring program, versus having staggered workshops spread over the two academic semesters*.* The sequence of the workshops was also found to be important, with mentors sharing the need to prioritize the Disability Awareness workshop.

## Discussion

A unique component of the pilot mentoring program was to enhance role-specific knowledge and skills for faculty and staff mentors, and student mentees with IDD, through the design and delivery of workshops. Evaluation of this unique workshop component was conducted by assessing knowledge acquisition pre- and post-tests at each workshop and surveys at the end of each semester. Findings from the program are discussed in the following section and have the potential to affect the formation of positive mentoring partnerships, future programs, and subsequently mentee outcomes.

### Areas of Knowledge Improvement

Faculty and staff mentors showed improvement in knowledge from before and after the Disability Awareness workshop. These findings indicate that mentors significantly improved in their knowledge of general disability awareness, such as using the correct terminology when speaking with someone with a disability, and having a greater understanding of invisible and intellectual disabilities. This is important as learning the appropriate form of language when talking with an individual with a disability can potentially improve the behavior and attitudes of mentees towards mentors (Feldman et al. 2002). Studies have also shown the need for mentors to be trained on encouraging mentees to talk about their disability, to dispel biases and assumptions (Rhodes et al. 2009), which can hamper the quality of the relationship (Daughtry et al. 2009). Having more knowledge of their disability can aid this process. Improved disability awareness will ultimately limit mentor bias, and thus enhance the potential for establishing positive and meaningful dyad relationships.

Faculty and staff mentors did not show improvements in knowledge after the workshops covering Communication, Program Basics, and Essential Mentor Skills, while student mentees did not demonstrate an increase in knowledge from before and after any of the four workshops they attended. This indicates that, on average, the responses were the same on the pre- and post-survey for these workshops. These non-significant findings may be the result of some program limitations inherent in a pilot study, which will be discussed below along with recommendations for future programs.

### Program Recommendations and Limitations

Results from this study indicate that for some workshops, mentors and mentees did not differ significantly in their responses on the pre- and post-test. There may be a few explanations for this. First, it is possible that for students with IDD the sentence structure and language used in the surveys were too complex. As a result, they may have consistently responded with the incorrect answer. Alternatively, some questions were perhaps too basic, and mentees responded with the correct answer both before and after the workshop. Finally, it is possible that student mentees may have simply lost interest in answering the same set of questions two hours apart and chose the first response option for both pre-posttests. While there is a lack of research identifying best practices for collecting data from students with IDD, mentoring programs looking to assess knowledge acquisition among students with IDD should look to use different methods of data collection (e.g., focus group sessions or interviews). In addition, attention should be paid to language and sentence construction to ensure student comprehension.

For mentors, on the other hand, content and questions on the workshop pre- and post-tests may have been too simplistic, or instructions unclear leading to no added knowledge acquisition. Some mentors, however, chose the incorrect answer on both pre-posttests, which indicates that future mentoring programs assessing knowledge acquisition must ensure that questions are clear and pertain to content specifically covered in the respective workshop. Additionally, it is possible that the small sample size for faculty and staff mentors who attended the workshops (15–18 pre- and post- respondents) may have also contributed to the null findings.

Next, findings from the qualitative data should also be incorporated to address changes to workshop content, style, and logistics. In particular, future mentoring programs should consider providing mentors access to workshops through a consolidated one-day training or as a series of webinars, before the first dyad meeting. Workshops related to disability awareness should be prioritized and expanded. In addition, mentors should be provided with information on the disability type of their mentee, specific strategies, and activities to utilize during dyad meetings, a list of available resources, recommended books to read, and a mentoring handbook.

This would lead to two benefits as suggested by the feedback from mentors: mentor participation in workshops may increase, and may also allow mentors to apply all the knowledge and skills from the outset. Keeping in mind the feedback for increased interaction, it would be conducive for future mentoring programs to schedule open forums for mentor discussions, and the opportunity for dyads to interact socially outside of the weekly meetings throughout the academic year. This is an essential component for building a trusting and meaningful mentoring relationship (Griffin et al. 2016; Jones and Goble 2012; McCallum 2018).

In-person workshops should be designed with more interactive methods of learning. It is evident that more hands-on, active methods are necessary, especially for mentees with IDD (Azar et al. 2016; Evmenova et al. 2017; Stavroussi et al. 2010) as it facilitates learning amongst this student population (Stavroussi et al. 2010; Wishart et al. 2007). Future mentoring programs should also ensure that workshop topics and content selected for the dyads align with the purpose of the mentoring program (such as employment and academic support for the EMP), and with the disability population they serve. For example, the decision to provide training on PFL, IFL, or both will have to be considered in light of current literature and group preferences.

Overall, feedback from both mentors and mentees highlight the benefit of workshop attendance. Having clear expectations about their respective roles, sufficient training, and expressing value in the workshops reinforces the need for future mentoring programs to incorporate dyad training. The EMP and the mixed methods used to evaluate the program can be undertaken by other organizations to support students with IDD navigate post-secondary education environments. However, additional research should be conducted on similar mentoring programs that address the above recommendations, using a larger sample size.

In summary, this paper describes the development, implementation, and evaluation of a pilot mentoring program designed to help students with IDD transition into a post-secondary education environment, and meet academic and career-related goals. Workshops were developed to enhance the role-specific knowledge and skills of both mentors and mentees, to build effective partnerships. While pre- and post-test findings demonstrated knowledge improvement among mentors in only one core mentoring concept, findings from the study highlighted areas for future improvement in respect to language and clarity of questions in surveys, increased interaction amongst mentors and dyads, online delivery of mentor workshops, enhancements to workshop content, and provision of additional resources for mentors. Changes informed by this feedback have the potential to optimize the mentoring experience and facilitate mentor and mentee learning.

Ultimately, the EMP demonstrates promise in helping students with IDD work towards academic and career-related goals, by providing mentorship training to both mentors *and* mentees. As such, other post-secondary institutions should look to implement mentoring programs that incorporate a formal training of dyads, which supports students with IDD through the transition into a post-secondary environment.
